# Cost‐effectiveness analysis of pembrolizumab plus chemotherapy with PD‐L1 test for the first‐line treatment of NSCLC

**DOI:** 10.1002/cam4.2793

**Published:** 2020-01-16

**Authors:** Ning Wan, Tian‐tian Zhang, Si‐hua Hua, Zi‐luo Lu, Bo Ji, Li‐xia Li, Li‐qing Lu, Wen‐jie Huang, Jie Jiang, Jian Li

**Affiliations:** ^1^ Department of Pharmacy General Hospital of Southern Theater Command Guangzhou Guangdong China; ^2^ Guangzhou Huabo Biopharmaceutical Research Institute Guangzhou Guangdong China; ^3^ College of Pharmacy Jinan University Guangzhou Guangdong China; ^4^ The First Affiliated Hospital of Jinan University Guangzhou Guangdong China; ^5^ International Cooperative Laboratory of Traditional Chinese Medicine Modernization and Innovative Drug Development of Chinese Ministry of Education (MOE) Jinan University Guangzhou Guangdong China; ^6^ Department of Oncology General Hospital of Southern Theater Command Guangzhou Guangdong China; ^7^ Department of Respiratory Medicine General Hospital of Southern Theater Command Guangzhou Guangdong China; ^8^ Dongguan Institute of Jinan University Dongguan China

**Keywords:** combination therapy, cost‐effectiveness, NSCLC, PD‐L1 test, pembrolizumab

## Abstract

**Background:**

Pembrolizumab (Pembro) in combination with chemotherapy has been approved for the treatment of pretreated advanced NSCLC in the United States and China for its significant efficacy. However, the cost‐effectiveness is unknown considering Pembro's high price. The impact of programmed death ligand 1 (PD‐L1) test on the cost‐effectiveness is also unknown. The current study assessed the cost‐effectiveness of combination therapy for nonsquamous NSCLC from the United States and China public payers’ perspective.

**Materials and Methods:**

A literature‐based Markov model was conducted using KEYNOTE‐189 trial data to compare cost and quality‐adjusted life years (QALYs) of three treatment strategies for nonsquamous NSCLC: Pembro‐chemotherapy combination and chemotherapy strategy without PD‐L1 test, and treatment strategy according to their PD‐L1 status.

**Results:**

In base case analysis, the combination strategy generated an additional 0.78 QALYs and 0.59 QALYs over chemotherapy in the United States and China respectively, resulting in an ICER of $132 392/QALY in the United States and $92 533/QALY in China. In the PD‐L1 ≥1% base case, the ICERs were $77 754/QALY and $56 768/QALY respectively in the United States and China for PD‐L1 test strategy. In the PD‐L1 ≥50% base case, the ICERs were $44 731/QALY and $34 388/QALY respectively in the United States and China for PD‐L1 test strategy. Lowering Pembro price can also partly decrease the ICERs.

**Conclusion:**

Compared with chemotherapy, the combination strategy is not cost‐effective for the treatment of NSCLC in the American and Chinese health care system at WTP threshold of $100 000/QALY for the United States and $27 351/QALY for China. Using PD‐L1 test for patient selection and price reduction could improve the cost‐effective probabilities of immunotherapy for nonsquamous NSCLC.

## Graphical abstract

A literature‐based Markov model was conducted using KEYNOTE‐189 trial data.The patient population was differentiated by PD‐L1 test.Different PD‐L1 positivity thresholds were applied to the model (1% or 50%).The combination strategy yielded superior health outcomes but not cost‐effective.Using PD‐L1 test for patient selection can improve the cost‐effective probability.

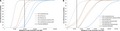

## 1 INTRODUCTION

Lung cancer always captures the world's attention for it accounts for nearly 20% cancer‐related deaths worldwide.^1^, ^2^, ^3^ The Global Burden of Disease Study reported that lung cancer has become one of the major causes of noncommunicable disease burden all over the world.^4^ In both the United States and China, lung cancer mortality ranked first among all cancers.^2^, ^5^, ^6^ In 2015, the total cost of lung cancer treatment in China reached ¥24.31 billion, accounting for 0.6% of the total health expenditure.^7^ And approximately 85% of lung cancer is nonsmall‐cell lung cancer (NSCLC).^8^ Nowadays, immune checkpoint inhibitor (ICI) has been a hot concern in oncology for its preferable therapeutic efficiency and safety.^9^, ^10^, ^11^, ^12^ Whether used as a monotherapy or in combination with chemotherapy, immunotherapy represents a new standard of care for advanced NSCLC in the frontline setting.^13^ Pembrolizumab (Pembro) is a monoclonal antibody against inhibitors of programmed death 1 (PD‐1), which exerts dual ligand blockade of the PD‐1 pathway, including programmed death ligand 1 (PD‐L1) and programmed death ligand 2 (PD‐L2), on antigen‐presenting or tumor cells. The combination of Pembro and standard chemotherapy (pemetrexed and a platinum‐based drug) has been approved by the United States Food and Drug Administration (FDA) May 2017^14^ and National Medical Products Administration (NMPA) in China in March 2019 as the first‐line therapy for patients with previously untreated metastatic nonsquamous NSCLC without EGFR or ALK mutations. Pembro combination therapy significantly longer overall survival (OS) and progression‐free survival (PFS) than chemotherapy alone (the estimated 12 months’ overall survival rate, 69.2% vs 49.4%, hazard ratio for death, 0.49, 95% CI: 0.38‐0.64, *P* < .001; the median progression‐free survival, 8.8 vs 4.9 months, hazard ratio for disease progression or death, 0.52, 95% CI: 0.43‐0.64, *P* < .001).^15^ In addition, it was reported that more survival benefits of Pembro‐chemotherapy combinations were observed in the subgroups of PD‐L1 tumor proportion score of 1%‐49% or ≥50% than those with PD‐L1 expression ＜1%.^15^ PD‐L1 expression is presumed to be a logical predictor of outcomes for immune checkpoint inhibitor therapies.^16^

Despite these enthusiastic results, high cost of emerging immunotherapy agents urge us to pay more attention to the need for pharmacoeconomic evaluation in order to guarantee the economic sustainability of health system and the access to care for all lung cancer patients.^16^, ^17^, ^18^ Therefore, our objective was to develop a cost‐effective evaluation to compare Pembro‐chemotherapy combination with chemotherapy alone in the United States and China, and to confirm whether it will be cost‐effective of using PD‐L1 expression to select the appropriate therapies for different patients.

## 2 MATERIALS AND METHODS

### 2.1 Model structure

**Figure 1:**
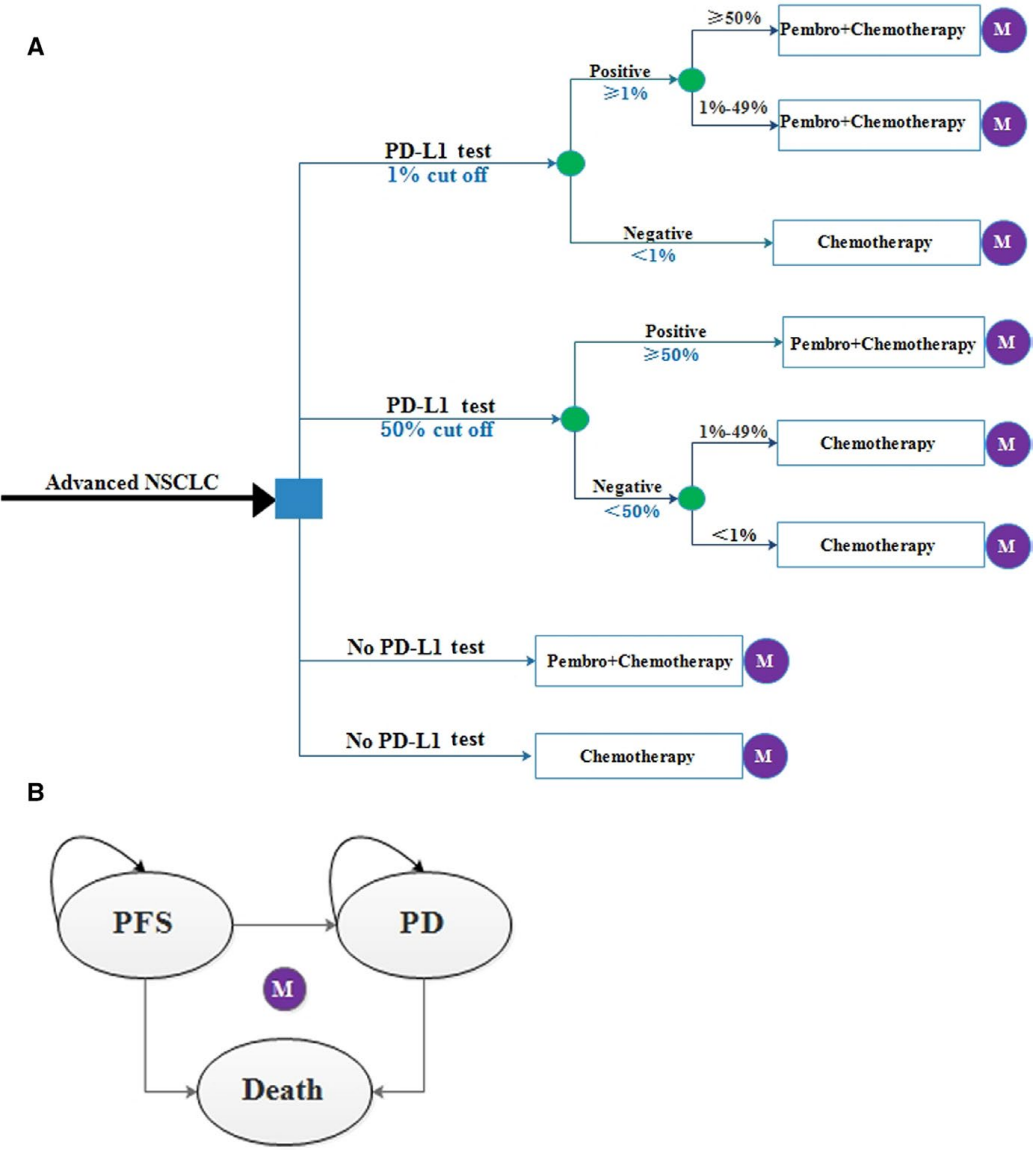
Model structure. A, The framework of the decision tree: PD‐L1 Test, patients tested for PD‐L1 expression; 1% cut off, a positive threshold of 1%; 50% cut off, a positivity threshold of 50%. B, The Markov state transition model. M, Markov node; NSCLC, non‐small cell lung cancer; PD, progression disease; PFS, progression‐free survival; Pembro, pembrolizumab

We constructed a literature‐based Markov models using clinical data from the available phase III study (KEYNOTE‐189) to compare cost and quality‐adjusted life years (QALYs) of three treatment strategies: (a) all patients treated with chemotherapy (pemetrexed and carboplatin/cisplatin) without tumor sample tested for PD‐L1 expression, (b) all patients treated with Pembro + Chemotherapy combination (pembrolizumab、pemetrexed and carboplatin/cisplatin) without tumor sample tested for PD‐L1 expression, and (c) patients treated according to their PD‐L1 status (PD‐L1 strategy): patients with PD‐L1‐positive status (a positivity threshold of 1% or 50%) were treated with Pembro + Chemotherapy combination, and patients with PD‐L1 negative status (below the positive threshold) were treated with chemotherapy (Figure 1A). In the primary analyses, Pembro + Chemotherapy was compared with Chemotherapy (PD‐L1 unselected base case), PD‐L1 test was compared with Chemotherapy (PD‐L1 ≥1% base case or PD‐L1 ≥50% base case), and PD‐L1 test was compared with Pembro + Chemotherapy. We calculated the incremental cost effectiveness ratios (ICERs) of all these comparisons for nonsquamous NSCLC patients in the United States and China. The PD‐L1 tumor proportion score was defined as the percentage of tumor cells with membranous PD‐L1 expression.

The target population was patients with previously untreated metastatic nonsquamous NSCLC without EGFR or ALK mutations. We conducted this economic evaluation from a public payer's perspective in the United States and China with a lifetime horizon to capture related costs and outcomes. Because all the regimens were administered every 3 weeks, a 3‐weeks cycle was used in the model. Cost of the PD‐L1 test, drug acquisition, major adverse events (AEs), treatments for progression, as well as injection and monitoring were considered to be direct medical costs. Drug prices in the United States were based on the 2019 Average Sales Price (ASP) data.^19^ Drug prices in China were estimated from the local bid‐winning price.^20^ Health utility values used for each health state in this model were obtained from the literature.^21^, ^22^, ^23^ Additionally, costs were discounted at an annual rate of 3%.^24^ All costs in China were converted into United States dollars using the exchange rate: 1 US dollar = 6.7 Chinese yuan renminbi. If the ICER is below $100 000 threshold in the United States or $27 351 threshold in China (three times of the per capita gross domestic product of China in 2017), the treatment is generally considered to be cost‐effective. Markov model was programmed using TreeAge Pro 2017 (TreeAge Software, Williamstown, Massachusetts) and R software (version 3.5.2) was applied to conduct additional statistical analyses.

### 2.2 Clinical inputs

The curves of progression‐free survival (PFS) and overall survival (OS) outcomes reported in the KEYNOTE‐189 trial were the sources of the effectiveness data for the models.^15^ Individual patient data were obtained by digitizing the Kaplan‐Meier survival curves reported in the trial and then fitted by Weibull or log‐logistic survival models using the method developed by Martin Hoyle and William Henley.^25^ For the validation purpose, we used r^2^ statistic to compare the predicted survival curves with the trial observed data(Table S1, Figures S1 and S2).^26^

The percentage of patients with PD‐L1 expression <1%, 1%‐49% and ≥50% were about 32.87%, 32.18%, and 34.95%, respectively (Table S1). Patients received either Pembro‐chemotherapy combination (n = 410, pembrolizumab (200 mg), pemetrexed (500 mg/m^2^), and carboplatin (AUC 5mg/mL/min)/cisplatin (75 mg/m^2^)) or chemotherapy (n = 206, pemetrexed (500 mg/m^2^), and carboplatin (AUC 5mg/mL/min)/cisplatin (75 mg/m^2^)) every 3 weeks for 4 cycles, followed by pembrolizumab‐pemetrexed combination (every 3 weeks for up to 35 cycles in the Pembro‐chemotherapy combination group) or pemetrexed (in the chemotherapy group).

After disease progression of the first‐line treatments, only 54.2% of patients in Pembro‐chemotherapy combination group received subsequent therapy in the KEYNOTE‐189 trial, hence we assumed the other 45.8% of patients were treated with supportive care. Crossover to Pembro monotherapy was permitted among the patients who received chemotherapy with verified disease progression. About 56.5% of patients in chemotherapy group were treated with subsequent therapy, and we assumed the others were treated with supportive care. All the patients received premedication with fosaprepitant、ondansetron, and glucocorticoids (dexamethasone) administered for carboplatin/cisplatin use; all the patients received premedication with folic acid, vitamin B12, and glucocorticoids(dexamethasone) administered for pemetrexed use; and all the patients received premedication with glucocorticoids(dexamethasone) administered for docetaxel use (Table S2).

### 2.3 Health care resource uses, costs, and utility

The costs of Pembro chemotherapy and other drugs in the United States were calculated based on the 2019 Average Sales Price (ASP) data,^19^ The sales price of each drug in China was estimated from the local bid‐winning price.^20^ Severe adverse events (top three in grade ≥ 3) were calculated in the economic evaluation because of their considerable impact on quality of life and health resource utilization, including anemia、neutropenia, and thrombocytopenia.^15^ The information of therapeutic regimen and AEs were collected from KEYNOTE‐189 trial. The United States costs of injection, administration, and AEs, as well as the cost of PD‐L1 test were adopted from published studies. In China, these costs were collected from Guangzhou Development and Reform Commission of China,^27^ local hospitals, and published studies. The utility values for metastatic nonsquamous NSCLC patients in the United States and China were cited from published study.^21^, ^22^, ^23^ The utility values of patients in PFS stage and PD stage were shown in Table 1. The utility of Pembro‐chemotherapy combination group and chemotherapy group was reasonably assumed to be the same, according to the similar incident chances of AEs that occurred between the two groups. The utility values were also slashedd with a 3% discount rate. All these data are listed in Table 1.^16^, ^19^, ^20^, ^21^, ^22^, ^23^, ^28^, ^29^, ^30^, ^31^, ^32^ For dosage calculation in the United States and China, body surface area (BSA) and body weight of 1.84 m^2^, 71.4 kg and 1.72 m^2^, 65 kg were adopted respectively.^33^, ^34^, ^35^

**Table 1:** Costs and health utilities

Parameters	United States			China		
	Value	Range	Ref	Value	Range	Ref
Price of pembrolizumab	48.987/1 mg	39.2‐58.8	19	26.74/mg^a^	21.4‐32.1	20
Price of pemetrexed	68.107/10 mg	54.5‐81.7	19	3.12/mg	2.5‐3.7	20
Price of carboplatin	3.252/50 mg	2.6‐3.9	19	0.16/mg	0.13‐0.19	20
Price of cisplatin	1.847/10 mg	1.5‐2.2	19	0.23/mg	0.18‐0.27	20
Price of nivolumab	27.498/1 mg	22 −33	19	13.82/mg	11.1‐16.6	20
Price of docetaxel	1.345/1 mg	1.1‐1.6	19	10.60/mg	8.5‐12.7	20
Monitoring cost per cycle	732	586‐878	16	102.5	82‐123	#
Cost for PD‐L1 test	60	48‐72	28	48.5	38.8‐58.2	#
Subsequent therapy cost per cycle						
Docetaxel	185.6	148‐223	19	1364	1092‐1638	20
Nivolumab	8835	7068‐10602	19	2689	21518‐32278	20
Pembrolizumab	9797	7838‐11756	19	5337	4270‐6404	20
Supportive care cost per cycle	3472	2778‐4166	28	338	159‐476	30
AEs managing cost per cycle						
Thrombocytopenia	1814	1451‐2177	29	6397	5117‐7676	31
Neutropenia	1043	834‐1251	29	466	415‐508	32
Anemia	1654	1323‐1985	29	537	478‐585	32
Utilities						
PFS	0.652	0.431‐0.833	23	0.804	0.536‐0.883	21, 22
PD	0.47	0.184‐0.773	23	0.321	0.05‐0.473	21, 22

Abbreviations: AEs, adverse events (grade > 3); PD, progression disease; PD‐L1 programmed death ligand 1; PFS, progression‐free survival.

a In China, eligible patients would pay for five cycles of pembrolizumab, followed by donations for five cycles of pembrolizumab (5 + 5); and then would pay for three cycles of pembrolizumab, followed by donations for three cycles of pembrolizumab; the donation (3 + 3) would continue until 24 months later or disease progression.

^#^ Data were collected from local hospitals.

### 2.4 Statistical analysis

The summary outcome was the ICER, calculated as the incremental cost per additional QALY gained between the treatment strategies under comparison. The main endpoint of this study was calculating ICERs for Pembro‐chemotherapy combination compared with chemotherapy, ICERs for PD‐L1 test compared with chemotherapy or Pembro‐chemotherapy combination.

We conducted a series of one‐way deterministic sensitivity analyses (DSA) to evaluate the influence of uncertainty in individual input variables on the ICER. All parameters were varied by adjusting within reported 95% confidence intervals (CI) or by assuming plausible ranges of the base case values (±20%) if none were available. We conducted a probabilistic sensitivity analysis (PSA) to test the stability of the model results. All the variables in the model were set in the distributions according to the recommendation of ISPOR‐SMDM Modeling Good Research Practices Task Force in conducting PSA.^36^ The variables about transition probabilities, proportion, and utility variables were assigned in beta distributions, and the variables about costs were assigned in gamma distributions. To simulate as many as possible situations, about 1000 kinds of random combination of different values (variables varied according to their preset distributions at the same time) were made. The result of PSA was presented as cost‐effectiveness acceptability curves that showed the probability of a strategy being cost‐effective compared with alternative treatment strategies on the basis of a willingness to pay (WTP) threshold of $100 000 and $27 351 per QALY gained used in the U.S and China respectively. Aside from base case analysis, two additional price reduction scenarios were taken into consideration to discover how the ICERs outcomes changed with the price of pembrolizumab in the United States reduced by 15% and 40%. One additional price reduction scenarios were taken into consideration with the price of pembrolizumab in China reduced by 50%. As in China, eligible patients could receive donated pembrolizumab from Chinese Primary Health Care Foundation: the patient would pay for five cycles of pembrolizumab, followed by donations for five cycles of pembrolizumab (5 + 5); and then would pay for three cycles of pembrolizumab, followed by donations for three cycles of pembrolizumab; the donation (3 + 3) would continue until 24 months later or until disease progression.

## 3 RESULTS

In the PD‐L1 unselected base case (Table 2), patients treated with Pembro‐chemotherapy combination provided a gain of 0. 78 QALYs and 0.59 QALYs over chemotherapy with an incremental cost of $102 870 and $54 565 in the United States and China, which resulted in an ICER of $132 392/QALY and $92 533/QALY respectively. In the PD‐L1 ≥1% base case, PD‐L1 test strategy provided a gain of 1.04 QALYs and 0.75 QALYs over chemotherapy strategy without PD‐L1 test with an incremental cost of $81 244 and $42 746 in the United States and China, resulting in an ICER of $77 754/QALY and $56 768/QALY respectively. In the PD‐L1 ≥50% base case, PD‐L1 test strategy provided a gain of 0.92 QALYs and 0.64 QALYs over the chemotherapy strategy without PD‐L1 test with an incremental cost of $41 250 and $22 009 in the United States and China, resulting in an ICER of $44 731/QALY and $34 388/QALY respectively. Either in the PD‐L1 ≥1% base case or in the PD‐L1 ≥50% base case, Pembro‐chemotherapy combination strategy without PD‐L1 test was weakly dominated by the PD‐L1 test strategy both in the United States and China (Table 2).

**Table 2:** Base case and scenario analyses

Treatment Arm	Mean Cost ($)	Effect QALY (Mean)	Compared with	Incremental Cost($)	Incremental Effect	ICER ($)
Base cases in the United States						
Chemotherapy	153 551	0.88				
Pembro + Chemotherapy	256 421	1.66	Chemotherapy	102 870	0.78	132 392
PD‐L1 test(1% cut off)	234 795	1.93	Chemotherapy	81 244	1.04	77 754
			Pembro + Chemotherapy	−21 626	0.27	Dominated
PD‐L1 test(50% cut off)	194 801	1.80	Chemotherapy	41 250	0.92	44 731
			Pembro + Chemotherapy	−61 620	0.14	Dominated
Price reduction scenarios in the United States						
Pembro cost reduced by 15%						
Chemotherapy	145 615	0.88				
Pembro + Chemotherapy	237 363	1.66	Chemotherapy	91 748	0.78	118 078
Pembro cost reduced by 40%						
Chemotherapy	132 388	0.88				
Pembro + Chemotherapy	205 600	1.66	Chemotherapy	73 211	0.78	94 222
Base cases in China						
Chemotherapy	61 072	0.78				
Pembro + Chemotherapy	115 637	1.37	Chemotherapy	54 565	0.59	92 533
PD‐L1 test(1% cutoff)	103 817	1.53	Chemotherapy	42 746	0.75	56 768
			Pembro + Chemotherapy	−11 820	0.16	Dominated
PD‐L1 test(50% cutoff)	83 081	1.42	Chemotherapy	22 009	0.64	34 388
			Pembro + Chemotherapy	−32 556	0.05	Dominated
Price reduction scenarios in China^a^						
Chemotherapy	46 942	0.78				
Pembro + Chemotherapy	81 934	1.37	Chemotherapy	34 992	0.59	59 340
PD‐L1 test(1% cutoff)	74 209	1.53	Chemotherapy	27 267	0.75	36 211
			Pembro + Chemotherapy	−7 725	0.16	Dominated
PD‐L1 test(50% cutoff)	61 011	1.42	Chemotherapy	14 070	0.64	21 983
			Pembro + Chemotherapy	−20 923	0.05	Dominated

Abbreviations: ICER, incremental cost‐effectiveness ratio; Pembro, pembrolizumab; QALY, quality adjusted life years.

a The cost of pembrolizumab was reduced by 50%. Chemotherapy, all patients treated with chemotherapy without tumor sample tested for PD‐L1 expression; Pembro + Chemotherapy, all patients treated with Pembro‐chemotherapy combination without tumor sample tested for PD‐L1 expression; PD‐L1 test(1% cutoff), patients with PD‐L1‐positive tumors (a positivity threshold of 1%) were treated with Pembro‐chemotherapy combination, and patients with PD‐L1‐negative tumors (below the positivity threshold) were treated with chemotherapy; PD‐L1 test(50% cutoff), patients with PD‐L1‐positive tumors (a positivity threshold of 50%) were treated with Pembro‐chemotherapy combination, and patients with PD‐L1‐negative tumors (below the positivity threshold) were treated with chemotherapy.

In the additional price reduction scenarios for PD‐L1 unselected base case in the United States (Table 2), 15% or 40% reduction of pembrolizumab price decreased the ICER to $118 078/QALY and $94,222/QALY respectively, resulting an acceptable ICER. As for the additional price reduction scenarios for PD‐L1 unselected base case in China, 50% reduction of pembrolizumab price decreased the ICER to $59 340/QALY. In the PD‐L1 ≥1% base case, PD‐L1 test strategy provided a gain of 0.75 QALYs over chemotherapy strategy without PD‐L1 test with an incremental cost of $27 267 in China by reducing pembrolizumab price by 50%, resuling in an ICER of $36 211/QALY. In the PD‐L1 ≥50% base case, PD‐L1 test strategy provided a gain of 0.64 QALYs over chemotherapy strategy without PD‐L1 test with an incremental cost of $14 070 and in China by reducing 50% of pembrolizumab price, resulting in an ICER of $21 983/QALY. Either in the PD‐L1 ≥1% base case or PD‐L1 ≥50% base case, Pembro‐chemotherapy combination strategy without PD‐L1 test was weakly dominated by the PD‐L1 test strategy both in China by reducing 50% of pembrolizumab price (Table 2).

**Figure 2:**
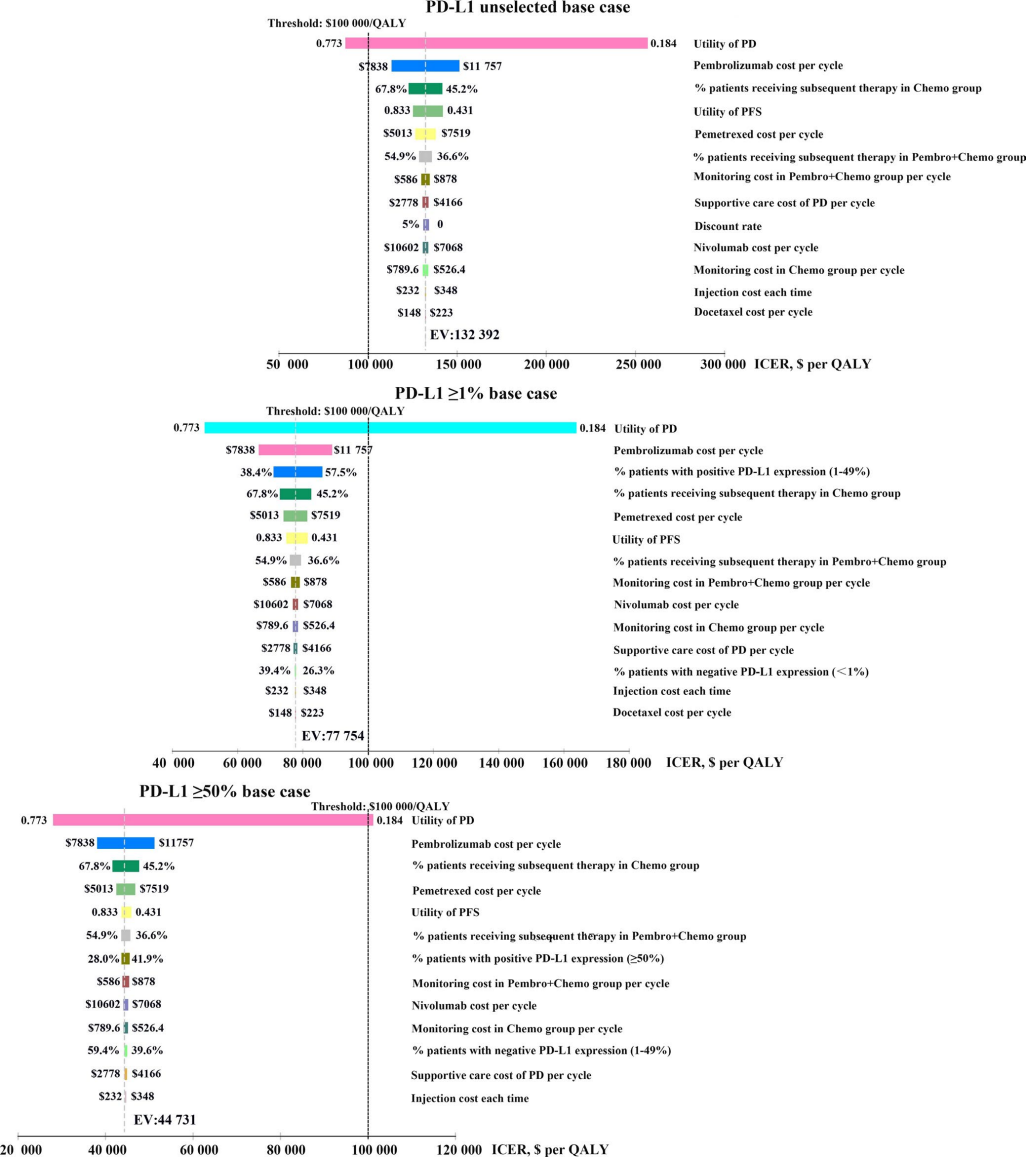
Tornado diagram of one‐way deterministic sensitivity analysis in the United States. PD‐L1 unselected base case: Pembro‐chemotherapy combination strategy without tumor sample tested for PD‐L1 expression vs chemotherapy strategy; PD‐L1 ≥1% base case: PD‐L1 test strategy with a positivity threshold of 1% vs chemotherapy strategy; PD‐L1 ≥50% base case: PD‐L1 test strategy (a positivity threshold of 50%) vs chemotherapy strategy

**Figure 3:**
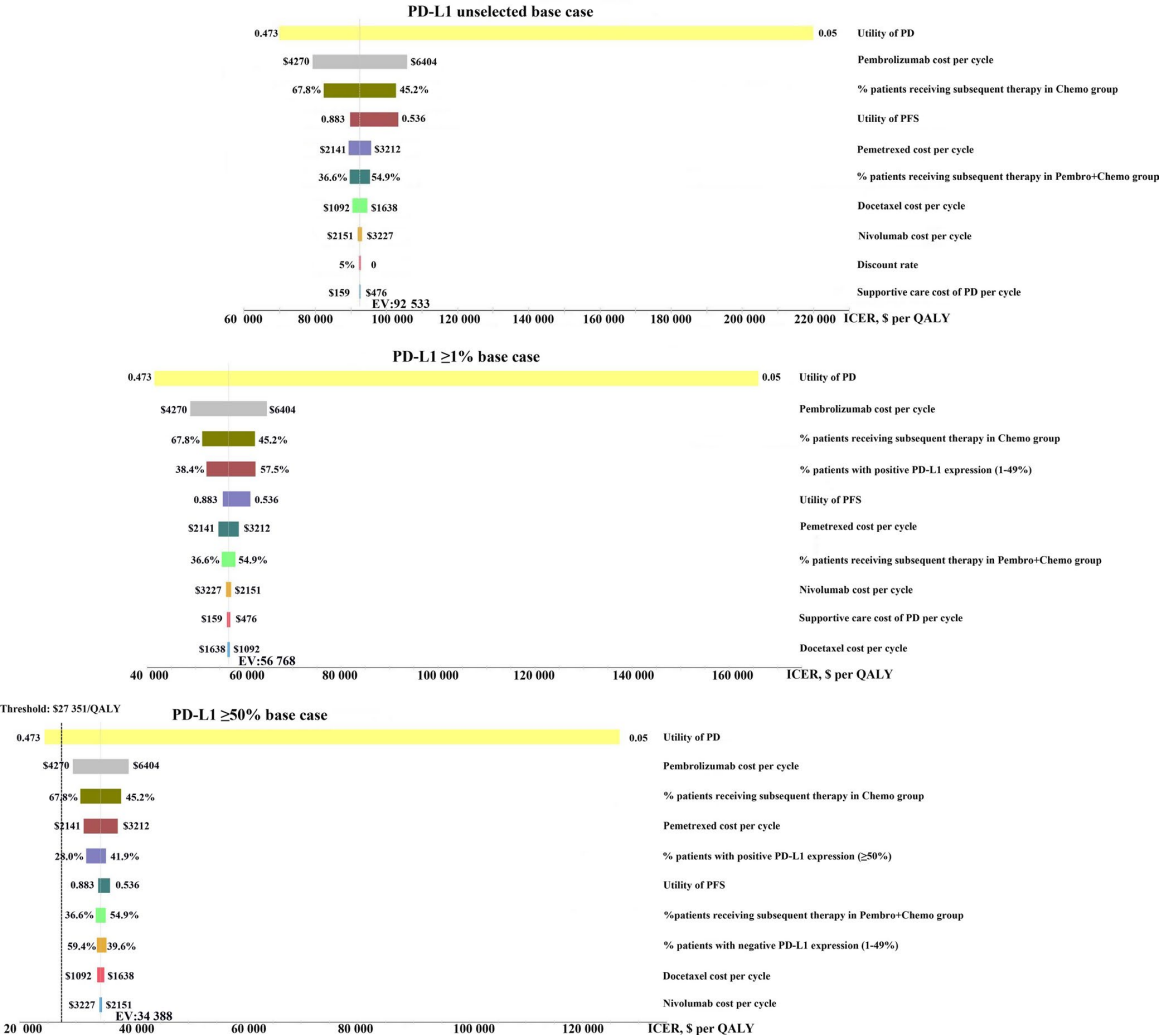
Tornado diagram of one‐way deterministic sensitivity analysis in China. PD‐L1 unselected base case: Pembro‐chemotherapy combination strategy without tumor sample tested for PD‐L1 expression vs chemotherapy strategy; PD‐L1 ≥1% base case: PD‐L1 test strategy with a positivity threshold of 1% vs chemotherapy strategy; PD‐L1 ≥50% base case: PD‐L1 test strategy (a positivity threshold of 50%) vs chemotherapy strategy

Tornado diagrams of one‐way sensitivity analyses are shown in Figure 2 (The United States) and Figure 3 (China). The results indicated that the utility of PD was the most sensitive parameter in the model, which had the greatest influence on the ICER. In the base case analyses including the PD‐L1 unselected, PD‐L1 ≥1% and PD‐L1 ≥50% base case, the combination strategy and PD‐L1 test strategy would become more cost effective as the utility of PD increased (Figures 2 and 3). In the PD‐L1 unselected base case both in the United States and China, the cost of pembrolizumab and pemetrexed, the proportion of patients receiving subsequent therapy in chemotherapy strategy or Pembro‐chemotherapy combination strategy, and the utility of PFS also had considerable impact on the ICER. In the PD‐L1 ≥1% base case, the cost of pembrolizumab and pemetrexed, the proportion of patients with PD‐L1 expression (1%‐49%), and the proportion of patients receiving subsequent therapy in chemotherapy strategy or Pembro‐chemotherapy combination strategy also had considerable impact on the ICER. In the PD‐L1 ≥50% base case, other than the above model inputs, the proportion of patients with PD‐L1 expression ≥50% also had considerable impact on the ICER. The ICERs of PD‐L1 test strategy vs Pembro‐chemotherapy combination strategy without PD‐L1 test turned out to be dominated when the parameters changed with default ranges in the United States (Figure S3). In the PD‐L1 ≥1% base case in China, the ICER of PD‐L1 test strategy vs Pembro‐chemotherapy combination strategy without PD‐L1 test was also dominated when the parameters changed with default ranges (Figure S4). While in the PD‐L1 ≥50% base case in China, the ICERs of PD‐L1 test strategy vs Pembro‐chemotherapy combination strategy without PD‐L1 test became unfavorable as the proportion of patients with PD‐L1 expression ≥50% and the utility of PD decreased (Supplementary Figure S4).

**Figure 4:**
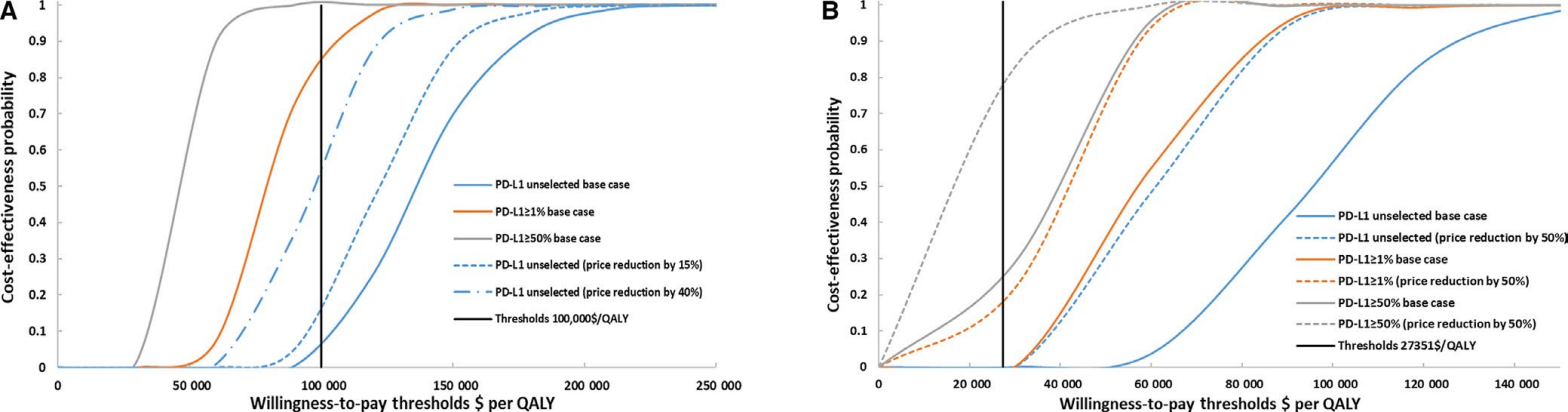
The cost‐effectiveness acceptability curves for base case analyses and price reduction scenarios in the United States and China

The cost‐effectiveness acceptability curves are shown in Figure 4. In the base case analyses, compared with the chemotherapy strategy, the Pembro‐chemotherapy combination strategy, PD‐L1 test strategy with a positivity threshold of 1% and 50% generated nearly 3.4%, 86.8%, and 100% cost‐effective probabilities when WTP was $100 000 in the United States (Figure 4A). Compared to the chemotherapy strategy, the Pembro‐chemotherapy combination strategy and PD‐L1 test strategy with a positivity threshold of ≥1% were not cost effective in the base case analyses in China, while PD‐L1 test strategy with a positivity threshold of 50% generated 18.5% probabilities of cost‐effectiveness (Figure 4B). In the price reduction scenarios, a price reduction of pembrolizumab by 15% and 40% in the United States, improved the cost‐effective probability of Pembro‐chemotherapy combination strategy to be 16.4% and 59.1% respectively (Figure 4A). With the price of pembrolizumab reduced by 50% in China, the Pembro‐chemotherapy combination strategy was still not cost‐effective, while the cost‐effective probability of PD‐L1 test strategy with a positivity threshold of ≥1% and 50% were improved and found to be 11.8% and 77.8%.

## 4 DISCUSSION

This evaluation investigated the cost‐effectiveness of pembrolizumab plus chemotherapy using PD‐L1 test to select Pembro‐chemotherapy combination therapy for patients with previously untreated metastatic nonsquamous NSCLC without EGFR or ALK mutation in the United States and China. Our results demonstrated that Pembro‐chemotherapy combination strategy generated an additional 0.78 QALYs and 0.59 QALYs over chemotherapy in the United States and China respectively, resulting in an ICER of $132 392/QALY in the United States and $92 533/QALY in China. At the WTP threshold values of $100 000/QALY and $27 351/QALY gained in the United States and China, these findings suggested that the Pembro‐chemotherapy combination strategy was not cost‐effective in the United States and China, which was also demonstrated by the acceptability curve with a paucity of certainty (Figure 4).

Until now, there is no relevant study in China for the pharmacoeconomic evaluation of this therapeutic regimen. Although a cost‐effectiveness analysis had been conducted from the U.S perspective,^37^ in which, the impact of PD‐L1 test on the cost‐effectiveness is unknown. In the current study, the patient population was differentiated by PD‐L1 test, and different PD‐L1 positivity thresholds were applied to the model (1% or 50%). Patients in different subgroups would receive different treatments, making therapy more targeted. In addition, although the establishment of models in both studies is based on KEYNOTE‐189, Markov model was designed herein, while partition model was utilized in the published study. In the published study, the result demonstrated that pembrolizumab in combination with chemotherapy yielded an ICER of $104 823/QALY, and our results are comparable to it. This study found that the Pembro + Chemotherapy gained additional 1.44 QALYs than chemotherapy. However, the current study only found additional 0.78 QALYs. One of the reasons for the substantial difference was because a different model was applied. Partitioned survival analysis model was developed to estimate the cost effectiveness of KEYNOTE‐189 trial comparators,^37^ while a Markov model was utilized in the current study. The second reason was the difference in the calculation of QALY to adopt the utility from patients receiving other treatments. Although the time to death utility of Pembro‐chemotherapy combination therapy for the treatment of NSCLC was reported in the Insinga's study,^37^ it did not distinguish the utility of PFS and PD.

PD‐L1, as a pembrolizumab's molecular target, is widely expressed at tumor cells surface which is often tested by IHC assay. In comparison with other molecular diagnostic tests for nonsquamous NSCLC, PD‐L1 expression test was less expensive and more available.^38^ Based on the NCCN guideline for NSCLC (2019, V2), the degree of recommendation of PD‐L1 detection is raised from 2A to level 1. The FDA has recently approved pembrolizumab monotherapy as the first‐line treatment for PD‐L1‐positive locally advanced/metastatic NSCLC, with PD‐L1 expression ≥1%, meanwhile without EGFR or ALK genomic aberrations based on KEYNOTE‐042 in April 2019.^39^ The previous recommendation level was PD‐L1 expression ≥50%.^40^ In May 2017 and August 2018, the FDA approved pembrolizumab in combination with pemetrexed plus carboplatin for the treatment of previously untreated, nonmutated, advanced NSCLC patients.^14^, ^41^ The approval of pembrolizumab in combination with chemotherapy as a first‐line treatment has also just been approved in China in March 2019. Also, using PD‐L1 test for selection of patients for immunotherapy seems to be an effective way to improve the cost‐effectiveness of the immunotherapy.^16^, ^38^ Hence in the current study, we attempted to use PD‐L1 test for the patients selecting of Pembro‐chemotherapy combination therapy, and two different positivity thresholds (1% or 50%) for PD‐L1 expression to the Markov model are adopted. In the PD‐L1 ≥1% base case, PD‐L1 test strategy produced nearly 86.8% cost‐effective probabilities over chemotherapy strategy in the United States, but the ICER was greater than the WTP threshold in China. In the PD‐L1 ≥50% base case, PD‐L1 test strategy produced nearly 100% and 18.5% cost‐effective probabilities over chemotherapy strategy in the United States and China, but the ICER was still greater than the WTP threshold in China. These results demonstrated that PD‐L1 test might improve the cost‐effective probabilities of Pembro‐chemotherapy combination therapy. Although PD‐L1 test strategy with a positivity threshold of 50% gained lower ICER than that with a positivity threshold of 1%, the latter one achieved higher QALY than the former one. This can be partly explained that patients treated with pembrolizumab plus chemotherapy with PD‐L1 expression of 1%‐49% also gained favorable effectiveness.^15^

Another way to improve cost‐effective probabilities is the reduction of drug prices. Through lowering the cost of pembrolizumab by 40%, Pembro‐chemotherapy combination strategy (without PD‐L1 test) vs chemotherapy produced an ICER below the United States WTP threshold with a 59.1% probability of cost effectiveness. Somehow, even the pembrolizumab price reduced by 50%, Pembro‐chemotherapy combination strategy (without PD‐L1 test) was still not cost‐effective in China. Under this circumstance, it is of great importance in using PD‐L1 test to select Pembro‐chemotherapy combination therapy for patients. With the price of pembrolizumab reduced by 50% in China, PD‐L1 test strategy (PD‐L1 ≥1% base case and PD‐L1 ≥50% base case) provided a gain of 0.75 QALYs and 0.64 QALYs over chemotherapy strategy, resulting in an ICER of $36,211/QALY and $21,983/QALY respectively, and the cost‐effective probability of PD‐L1 test strategy with a positivity threshold of ≥1% and 50% were improved to be 11.8% and 77.8%.

There are also several limitations in the current analysis that deserve consideration. Firstly, the final conclusions about cost‐effectiveness largely depend on the WTP threshold of the country, which often ranges between $100 000/QALY and $150 000/QALY in the United States,^42^ and this study was approximately $100 000/QALY at the low end of the United States WTP. Secondly, although utility being the dominant factor affecting the ICER according to sensitivity analysis, there is no specific utility data available for the Markov model since no related research had been constructed to estimate the utility of patients who have received Pembro‐chemotherapy combination therapy for the treatment of NSCLC. There is inevitably a bias in the calculation of QALY to adopt the utility from patients receiving other treatments. Thirdly, as the disease progresses, due to ethical requirements, most patients who had previously received chemotherapy may cross over to pembrolizumab treatment, leading to deviation in the calculation of ICER.

## 5 CONCLUSIONS

In conclusion, compared with chemotherapy, Pembro‐chemotherapy combination therapy is not cost‐effective in the United States and China health care system for the treatment of nonsquamous NSCLC at a WTP threshold of $100 000/QALY for the United States and $27 351/QALY for China. Using PD‐L1 test for patient selection is worth the consideration in nonsquamous NSCLC patients to improve the cost‐effective probabilities of immunotherapy.

## CONFLICT OF INTEREST

The authors declare that they have no conflicts of interest.
