# Supplementary material for: Cost‐effectiveness analysis of pembrolizumab plus chemotherapy with PD‐L1 test for the first‐line treatment of NSCLC
Source: Cancer Med. 2020 Jan 16;9(5):1683–93. doi: 10.1002/cam4.2793 (PMC7050096; doi:10.1002/cam4.2793)
Supplement: Supplementary file 1 [file CAM4-9-1683-s001.docx]

**Supplementary Material**

Table S1. Clinical inputs to the models

| **Parameter** | **Values** | | | **Ref** |
| --- | --- | --- | --- | --- |
| **Weibull OS survival model** |  |  |  |  |
| 1-st line pembrolizumab-chemotherapy combination(Whole Patients) | lambda=0.01021335 | gamma= 1.274085 | r^2^=0.979 | [1] |
| 1-st line pembrolizumab-chemotherapy combination(Tumor Proportion Score of <1%) | / | / | / | / |
| 1-st line pembrolizumab-chemotherapy combination(Tumor Proportion Score of 1-49%) | lambda =0.01376783 | gamma =1.099352 | r^2^=0.952 | [1] |
| 1-st line pembrolizumab-chemotherapy combination(Tumor Proportion Score of ≥50%) | lambda=0.02952912 | gamma=0.7721453 | r^2^=0.955 | [1] |
| 1-st line chemotherapy(Whole Patients) | lambda=0.02052450 | gamma=1.26675004 | r^2^=0.982 | [1] |
| 1-st line chemotherapy(Tumor Proportion Score of <1%) | lambda=0.03149349 | gamma=1.14424451 | r^2^=0.978 | [1] |
| 1-st line chemotherapy(Tumor Proportion Score of 1-49%) | theta =-3.965 | kappa =1.51236 | r^2^=0.951 | [1] |
| 1-st line chemotherapy(Tumor Proportion Score of ≥50%) | / | / | / | / |
| **Weibull PFS survival model** |  |  |  |  |
| 1-st line pembrolizumab-chemotherapy combination(Whole Patients) | lambda=0.02913568 | gamma=1.26022049 | r^2^=0.992 | [1] |
| 1-st line pembrolizumab-chemotherapy combination(Tumor Proportion Score of <1%) | / | / | / | / |
| 1-st line pembrolizumab-chemotherapy combination(Tumor Proportion Score of 1-49%) | lambda=0.02891478 | gamma=1.20905529 | r^2^=0.981 | [1] |
| 1-st line pembrolizumab-chemotherapy combination(Tumor Proportion Score of ≥50%) | lambda=0.02262898 | gamma=1.26308924 | r^2^=0.978 | [1] |
| 1-st line chemotherapy combination(Whole Patients) | lambda=0.04925208 | gamma=1.26167310 | r^2^=0.989 | [1] |
| 1-st line chemotherapy(Tumor Proportion Score of <1%) | lambda=0.04254582 | gamma=1.39990251 | r^2^=0.988 | [1] |
| 1-st line chemotherapy(Tumor Proportion Score of 1-49%) | lambda=0.03123310 | gamma=1.42975214 | r^2^=0.962 | [1] |
| 1-st line chemotherapy(Tumor Proportion Score of ≥50%) | / | / | / | / |
| **Grade 3+ Adverse Events** |  |  |  |  |
| Anaemia happened in pembrolizumab-chemotherapy combination group | Risk of Hospitalizationa=16.30% | Risk of Hospitalization(per cycle)=4.35% | costs(per cycle)=1654 | [1,2] |
| Neutropenia happened in pembrolizumab-chemotherapy combination group | Risk of Hospitalizationa=15.80% | Risk of Hospitalization(per cycle)=4.21% | costs(per cycle)=1043 | [1,2] |
| Thrombocytopenia happened in pembrolizumab-chemotherapy combination group | Risk of Hospitalizationa=7.90% | Risk of Hospitalization(per cycle)=2.04% | costs(per cycle)=1814 | [1,2] |
| Anaemia happened in chemotherapy group | Risk of Hospitalizationa=15.35% | Risk of Hospitalization(per cycle)=4.08% | costs(per cycle)=1654 | [1,2] |
| Neutropenia happened in chemotherapy group | Risk of Hospitalizationa=11.88% | Risk of Hospitalization(per cycle)=3.11% | costs(per cycle)=1043 | [1,2] |
| Thrombocytopenia happened in chemotherapy group | Risk of Hospitalizationa=6.93% | Risk of Hospitalization(per cycle)=1.78% | costs(per cycle)=1814 | [1,2] |
| **PD-L1 expression** |  |  |  |  |
| **PD-L1 tumor proportion score — no. (%)** |  |  |  |  |
| <1% in pembrolizumab-chemotherapy combination group | 127(32.8) |  |  | [1] |
| ≥1% in pembrolizumab-chemotherapy combination group | 260(67.2) |  |  | [1] |
| 1–49% in pembrolizumab-chemotherapy combination group | 128(33.0) |  |  | [1] |
| ≥50% in pembrolizumab-chemotherapy combination group | 132(34.2) |  |  | [1] |
| <1% in chemotherapy group | 63(33.0) |  |  | [1] |
| ≥1% in chemotherapy group | 128(67.0) |  |  | [1] |
| 1–49% in chemotherapy group | 58(30.4) |  |  | [1] |
| ≥50% in chemotherapy group | 70(36.6) |  |  | [1] |
| **PD-L1–positive tumors (a positivity threshold of ≥1%)** | 32.87% | distribution:triangular |  | [1] |
| **the proportion of PD-L1 expression 1-49% in PD-L1–positive tumors (a positivity threshold of ≥1% )** | 47.94% | distribution:triangular |  | [1] |
| **PD-L1–negative tumors (a positivity threshold of≥50%)** | 34.95% | distribution:triangular |  | [1] |
| **the proportion of PD-L1 expression 1-49% in PD-L1–negative tumors (a positivity threshold of≥50%)** | 49.47% | distribution:triangular |  | [1] |
| **treated with Carboplatin in PFS** |  |  |  |  |
| pembrolizumab-chemotherapy combination group | 73% |  |  | [1] |
| chemotherapy group | 72% |  |  | [1] |
| **treated with Cisplatin in PFS** |  |  |  |  |
| pembrolizumab-chemotherapy combination group | 27% |  |  | [1] |
| chemotherapy group | 28% |  |  | [1] |
| **therapy in PD** |  |  |  |  |
| **Any subsequent therapy in PD in pembrolizumab-chemotherapy combination group** | 46% | distribution:triangular |  | [1] |
| treated with Docetaxel in pembrolizumab-chemotherapy combination group | 88% |  |  |  |
| treated with Nivolumab in pembrolizumab-chemotherapy combination group | 6% |  |  | [1] |
| treated with Pembrolizumab in pembrolizumab-chemotherapy combination group | 6% |  |  | [1] |
| **supportive care in pembrolizumab-chemotherapy combination group** | 54% |  |  |  |
| **Any subsequent therapy in PD in chemotherapy group** | 56% | distribution:triangular |  |  |
| treated with Docetaxel in chemotherapy group | 14% |  |  |  |
| treated with Nivolumab in chemotherapy group | 15% |  |  | [1] |
| treated with Pembrolizumab in chemotherapy group | 72% |  |  | [1] |
| **supportive care in chemotherapy group** | 44% |  |  | [1] |
| **Discount rate** | 3%(range:0–5%) |  |  |  |

Table S2. Pre-medication Costs

| **Pre-medications** | **Strength** | **^#^ of doses** | **Average cost per cycle** | **Ref** |
| --- | --- | --- | --- | --- |
| **In cycles containing carboplatin** |  |  | **321** |  |
| Fosaprepitant (anti-emetic) | 150 mg | 1 | 315.60 | [3,4] |
| Ondansetron (anti-emetic) | 24 mg | 1 | 4.08 | [3,4] |
| Dexamethasone (steroid) | 4 mg | 3 | 1.30 | [3,4] |
| **In cycles containing cisplatin** |  |  | **324** |  |
| Fosaprepitant (anti-emetic) | 150 mg | 1 | 315.60 | [3,4] |
| Dexamethasone (steroid) | 4 mg | 13^a^ | 5.62 | [3,4] |
| Ondansetron (anti-emetic) | 8 mg | 2 | 2.72 | [3,4] |
| **In cycles containing pemetrexed** |  |  | **4** |  |
| folic acid | 400 μg | 21^b^ | 0.48 | [3,4] |
| Vitamin B12 injection^c^ | 1000 μg | 1/3 | 0.58 | [3,4] |
| Dexamethasone (steroid) | 4 mg | 6^d^ | 2.59 | [3,4] |
| **In cycles containing Docetaxel** |  |  | **5** |  |
| Dexamethasone (steroid) | 8 mg | 6^d^ | 5.18 | [3,4] |

# :^a^3 doses on day 1, 2 on day 2, and 4 each on days 3-4; ^b^1 dose every day; ^c^Administered every 3rd cycle that pemetrexed is given; ^d^2 doses on day 1,2,the day before

Table S3. Parametric survival distributions fitted for the various study regimens.

| Parametric Model | Pembro+Chemotherapy (without PD-L1 test) | | Pembro+Chemotherapy (PD-L1:1%~49%) | | Pembro+Chemotherapy (PD-L1 ≥50%) | | Chemotherapy (without PD-L1 test) | | Chemotherapy (PD-L1<1%) | | Pembro+Chemotherapy (PD-L1:1%~49%) | |
| --- | --- | --- | --- | --- | --- | --- | --- | --- | --- | --- | --- | --- |
|  | AIC | BIC | AIC | BIC | AIC | BIC | AIC | BIC | AIC | BIC | AIC | BIC |
| PFS |  |  |  |  |  |  |  |  |  |  |  |  |
| Weibull | 1840.4 | 1848.5 | 550.2 | 555.8 | 531.8 | 537.4 | 1051.8 | 1058.4 | 336.8 | 341.0 | 287.2 | 291.4 |
| Log-logistic | 1844.8 | 1852.8 | 548.2 | 553.9 | 536.2 | 541.9 | 1053.3 | 1060.0 | 339.4 | 343.6 | 285.1 | 289.2 |
| Exponential | 1854.0 | 1862.0 | 553.2 | 558.9 | 535.6 | 541.2 | 1062.8 | 1069.4 | 344.2 | 348.5 | 293.9 | 298.0 |
| Logistic | 1954.0 | 1962.0 | 590.7 | 596.4 | 554.7 | 560.4 | 1128.9 | 1135.5 | 356.5 | 360.7 | 303.6 | 307.8 |
| OS |  |  |  |  |  |  |  |  |  |  |  |  |
| Weibull | 1240.7 | 1248.8 | 419.6 | 425.5 | 380.2 | 386.2 | 885.3 | 892.0 | 250.4 | 254.7 | 250.4 | 254.7 |
| Log-logistic | 1243.4 | 1251.4 | 418.0 | 423.8 | 383.0 | 389.0 | 880.8 | 887.5 | 249.5 | 253.8 | 249.5 | 253.8 |
| Exponential | 1241.7 | 1250.8 | 420.0 | 425.8 | 384.8 | 390.8 | 887.1 | 893.8 | 252.0 | 256.4 | 252.0 | 256.4 |
| Logistic | 1327.7 | 1335.7 | 466.0 | 471.8 | 429.5 | 435.5 | 956.1 | 962.8 | 265.1 | 269.4 | 265.1 | 269.4 |

AIC = Akaike information criterion; BIC = Bayesian information criterion; OS = overall survival; PFS = pro- gression-free survival; Pembro =pembrolizumab


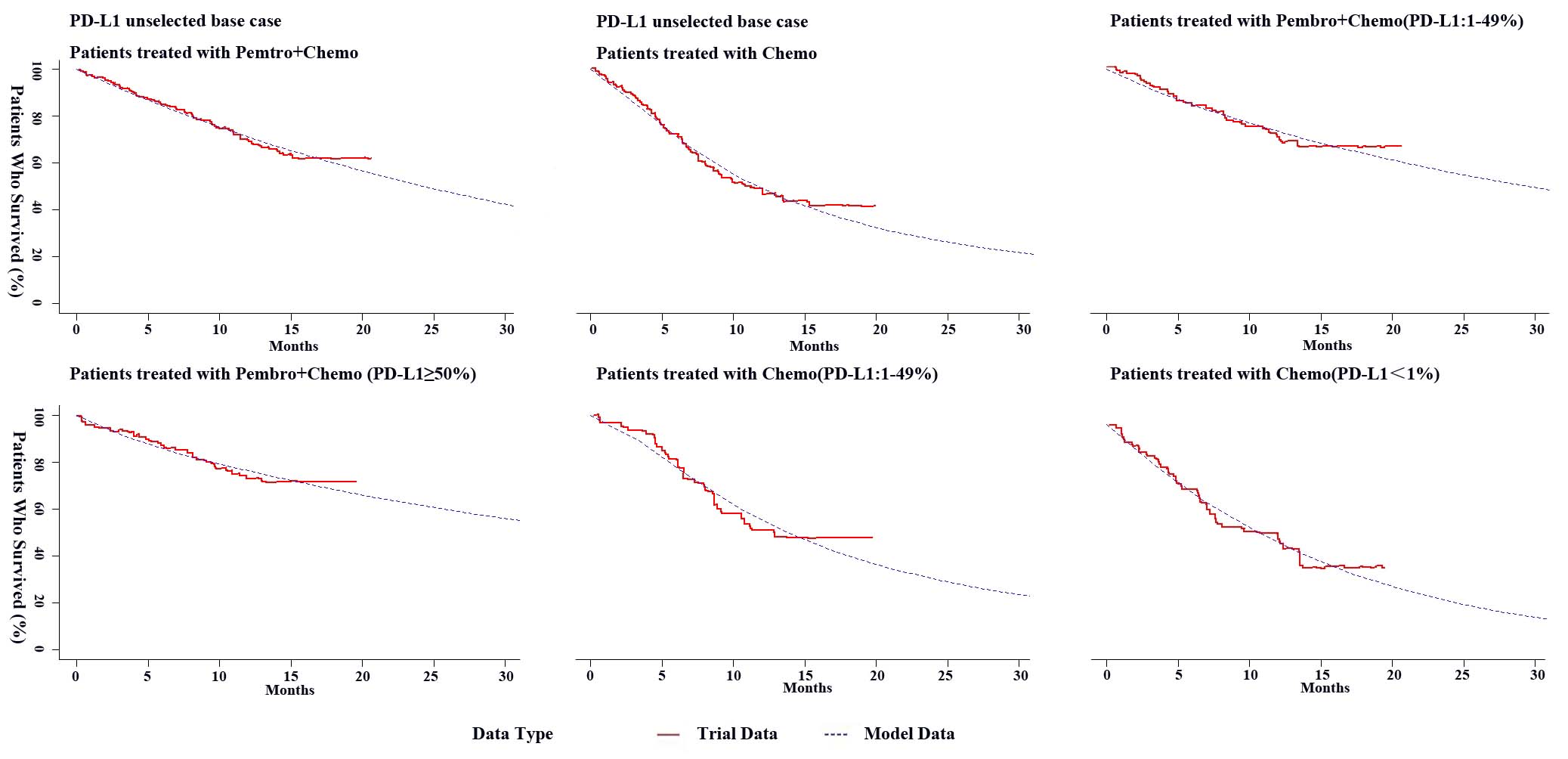


Figure S1. KM Data and Extrapolated ToT Based on Weibull/ LogLogistic Distribution for Overall Survival. KM, Kaplan-Meier; ToT, time on treatment.


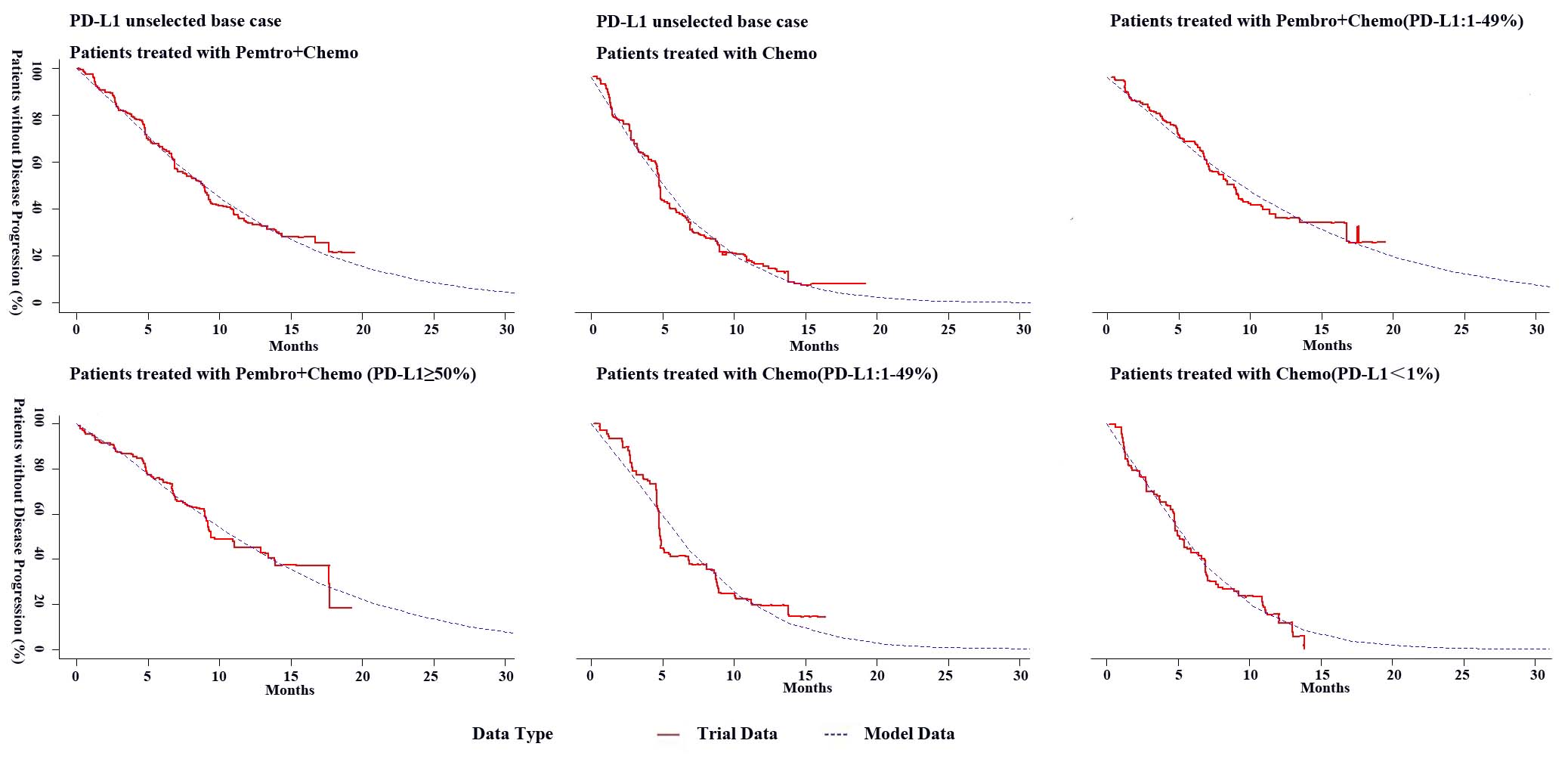


Figure S2. KM Data and Extrapolated ToT Based on Weibull/ LogLogistic Distribution for Progression-free Disease. KM, Kaplan-Meier; ToT, time on treatment.

**
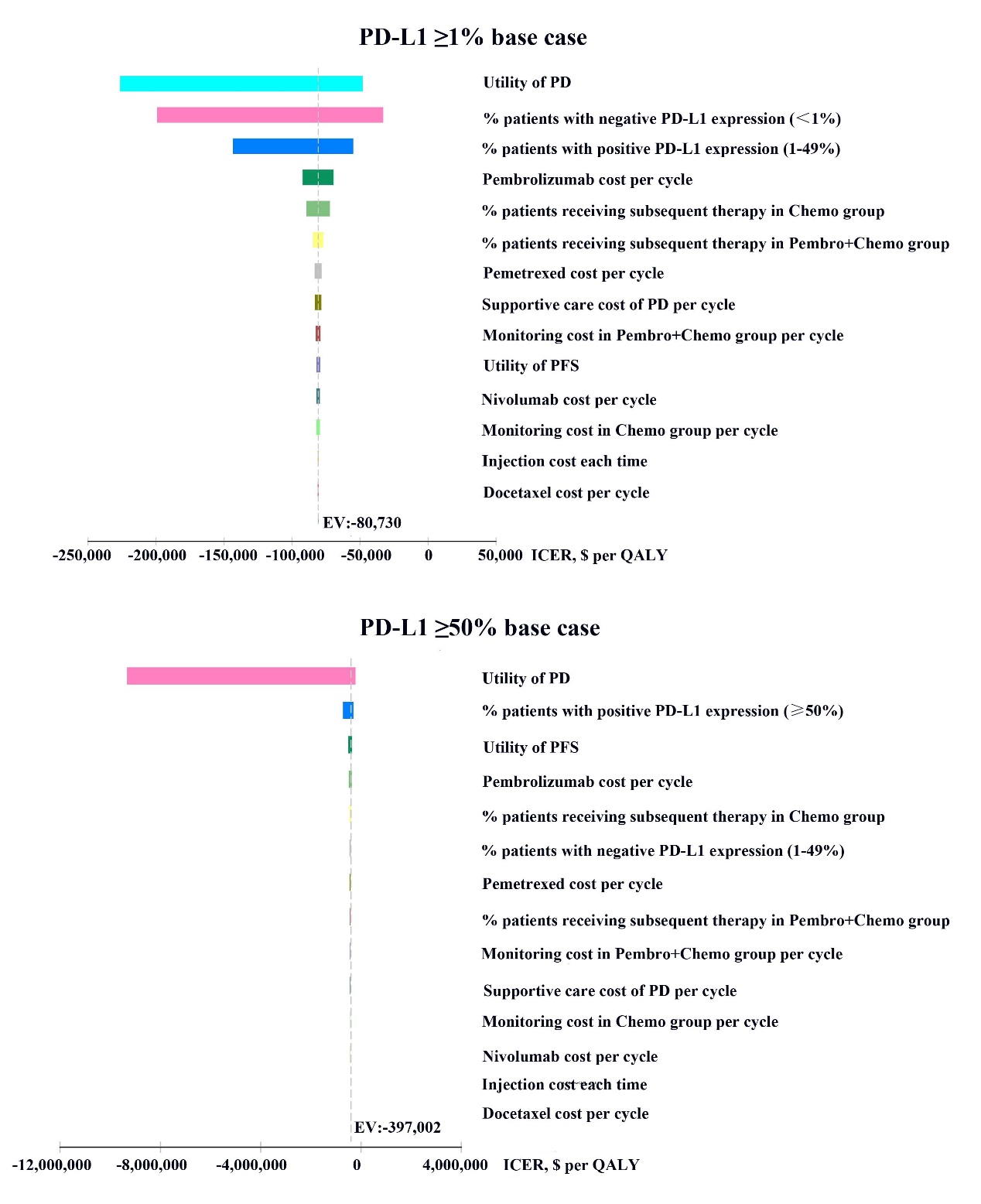
**

Figure S3. Tornado diagram of one-way deterministic sensitivity analysis in the US. PD-L1≥1% base case: PD-L1 test strategy with a positivity threshold of 1% versus pembrolizumab plus chemotherapy strategy; PD-L1≥50% base case: PD-L1 test strategy (a positivity threshold of 50%) versus pembrolizumab plus chemotherapy strategy.

**
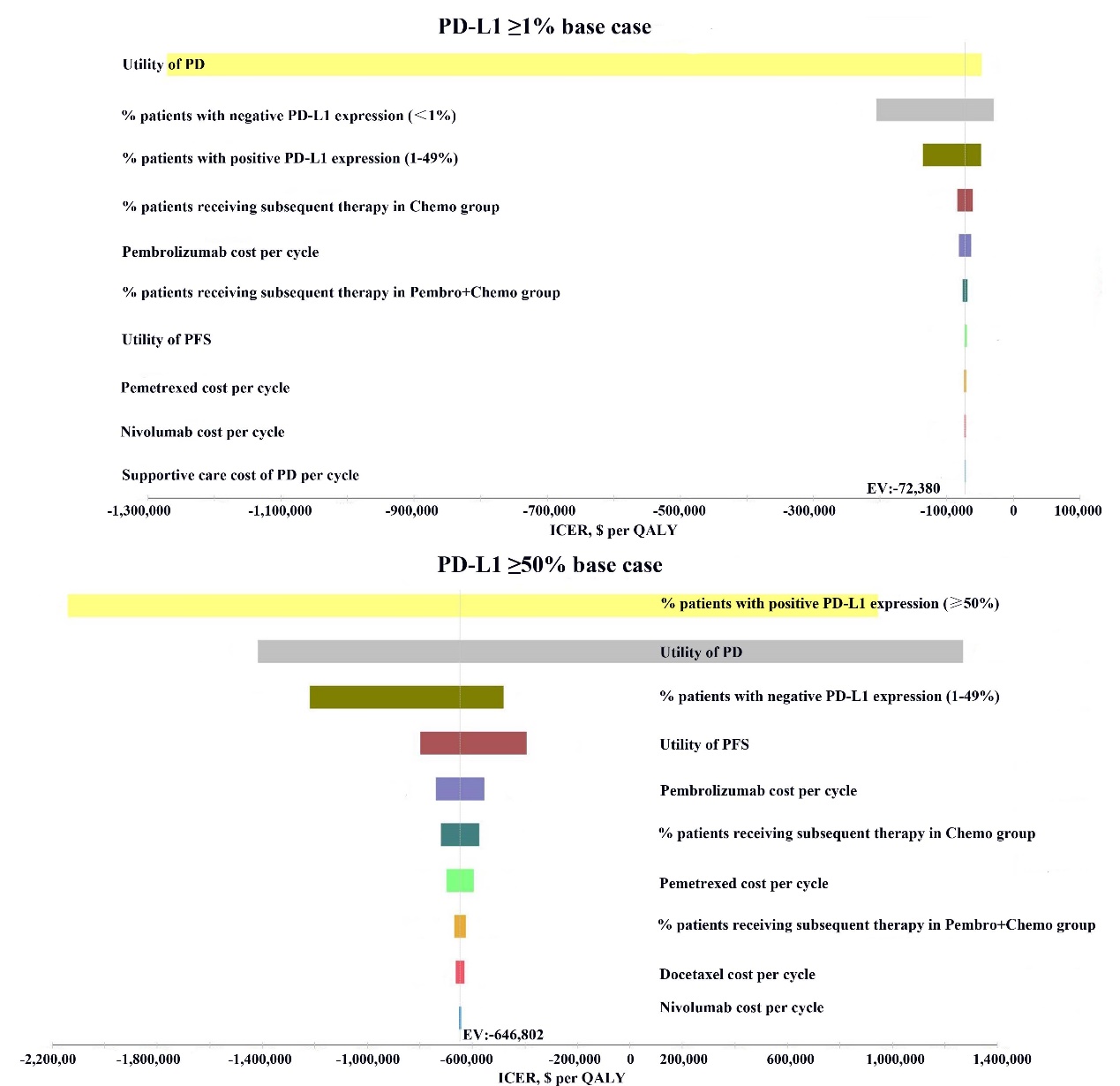
**

Figure S4 Tornado diagram of one-way deterministic sensitivity analysis in China. PD-L1≥1% base case: PD-L1 test strategy with a positivity threshold of 1% versus pembrolizumab plus chemotherapy strategy; PD-L1≥50% base case: PD-L1 test strategy (a positivity threshold of 50%) versus pembrolizumab plus chemotherapy strategy.

**The highest top three grade 3+ adverse events were taken into account.**

Data from Clinical trial Keynote-189 including serious adverse events that occurred within 90 days. For simplicity, only the highest top three grade 3+ adverse events happened in pembrolizumab-chemotherapy combination group/chemotherapy group (Anaemia: 16.30%/ 15.35%; Neutropenia: 15.80%/ 11.88%; Thrombocytopenia: 7.90%/ 6.93%) were taken into account in this study. According to TreeAgePro2011 operation manual, the rates of adverse events were transformed by formulas as follows: r=[ln(1-P)]/t; P=1-e^(-rt) [5]. As the results, Anaemia,Neutropenia, Thrombocytopenia happened 4.35%/ 4.08%, 4.21%/ 3.11%, 2.04%/ 1.78% in pembrolizumab-chemotherapy combination group/chemotherapy group per(per cycle), respectively. And the costs of these adverse events were renewed from the published resources [2].

**The percentage of patients who receive subsequent therapy after disease progression was estimated from the KN189 trial.**

Data from Clinical trial Keynote-189 including specific subsequent schemes patients receives after disease progression. There were 46% and 56% patients received specific subsequent schemes in pembrolizumab-chemotherapy combination group and chemotherapy group, respectively [1]. Therefore, we assumed the other 54% and 44% patients received supportive care in pembrolizumab-chemotherapy combination group and chemotherapy group, respectively. For simplicity, only the specific subsequent schemes used by at least 5% of patients and schemes of immune checkpoint inhibitor for its’ significance impactions in costs in each trial arm are explicitly modeled. Docetaxel, nivolumab monotherapy, pembrolizumab monotherapy represented the specific subsequent schemes patients receives after disease progression. For it was reported in Clinical trial Keynote-189 about 2.9% and 2.6% of patients in pembrolizumab-chemotherapy combination group received nivolumab monotherapy and pembrolizumab monotherapy in PD, respectively, and about 8.2% and 40.6% of patients in chemotherapy group received nivolumab monotherapy and pembrolizumab monotherapy in PD, respectively [1]. We assumed the percentage of nivolumab monotherapy and pembrolizumab monotherapy(6% and 6% of the specific subsequent schemes respectively in pembrolizumab-chemotherapy combination group, and 15% and 72% of the specific subsequent schemes respectively in chemotherapy group) as them actually were, the rest of the specific subsequent schemes, 88% in pembrolizumab-chemotherapy combination group and 14% in chemotherapy group was Docetaxel.

**Reference**

[1] Gandhi L, Rodríguez-Abreu D, Gadgeel S, et al. Pembrolizumab plus chemotherapy in metastatic non–small-cell lung cancer. *N Engl J Med*, 2018, 378(22): 2078-2092.

[2] Centers for Disease Control and Prevention. <https://www.cms.gov/Medicare/Medicare-Fee-for-Service-Payment/AcuteInpatientPPS/FY2018-IPPS-Final-Rule-Home-Page.html>

[3] Insinga RP, Vanness DJ, Feliciano JL, et al. Cost-effectiveness of pembrolizumab in combination with chemotherapy in the 1st line treatment of non-squamous NSCLC in the U.S.[J]. J Med Genet, 2018:1-28.

[4] Centers for Disease Control and Prevention. 2019 ASP drug pricing files. https://www.cms.gov/apps/ama/license.asp?file=/Medicare/Medicare-Fee-for-Service-Part-B-Drugs/McrPartBDrugAvgSalesPrice/downloads/April-2019-ASP-Pricing-File.zip (accessed April 14, 2019).

[5] Hoyle MW, Henley W. Improved curve fits to summary survival data: application to economic evaluation of health technologies. BMC Med Res Methodol. 2011; 11:139.
